# I-131-Metaiodobenzylguanidine therapy with allogeneic cord blood stem cell transplantation for recurrent neuroblastoma

**DOI:** 10.1186/1824-7288-38-53

**Published:** 2012-10-15

**Authors:** Yuya Sato, Hidemitsu Kurosawa, Keitaro Fukushima, Mayuko Okuya, Susumu Hagisawa, Kenichi Sugita, Osamu Arisaka, Anri Inaki, Hiroshi Wakabayashi, Ayane Nakamura, Makoto Fukuoka, Daiki Kayano, Seigo Kinuya

**Affiliations:** 1 Department of Pediatrics, Dokkyo Medical University School of Medicine, 880 Kita-Kobayashi, Mibu, Tochigi, Japan, 321-0293; 2 Department of Nuclear Medicine, Kanazawa University, Kanazawa, Ishikawa, Japan

**Keywords:** MIBG, Neuroblastoma, Allogeneic cord blood stem cell transplantation

## Abstract

Iodine-131-metaiodiobenzylguanidine (^131^I-MIBG) therapy combined with allogeneic cord blood stem cell transplantation (SCT) was used to treat a 4-year-old girl with recurrent neuroblastoma. The patient experienced relapse 2 years after receiving first-line therapies, which included chemotherapy, surgical resection, irradiation, and autologous peripheral SCT. Although ^131^I-MIBG treatment did not achieve complete remission, the size of the tumor was reduced after treatment. Based on our findings, we suggest that ^131^I-MIBG treatment with myeloablative allogeneic SCT should be considered as first-line therapy for high-risk neuroblastoma patients when possible.

## Background

Neuroblastoma is the most common extracranial solid tumor of childhood. Although dose-intensive treatments have improved the prognosis of patients with advanced neuroblastoma, the prognosis of this disease remains very poor, and the survival rate is estimated to be 25% [1]. Myeloablative allogeneic stem cell transplantation (SCT) rescue has not been extensively used to treat neuroblastoma, primarily because of the toxicity of megatherapy regimens and the risk of graft-versus-host disease (GVHD) [2,3].

Recent reports have described the use of iodine-131-metaiodiobenzylguanidine (^131^I-MIBG) treatment combined with autologous SCT rescue in patients with recurrent neuroblastoma; however, this treatment does not always yield satisfactory results [4,5].

## Case report

The subject was a 2-year-old female with a diagnosis of stage 4 neuroblastoma with unfavorable histology according to the international neuroblastoma pathology classification, non-amplification of MYCN, and primary localization to the left adrenal gland with metastasis to the thoracic vertebrae, pelvis, and bone marrow. Vanillylmandelic acid (VMA) was elevated to 448.2 μg/mg creatinine (Cr) and homovanillic acid (HVA) was increased to 127.8 μg/mg Cr. The patient was treated with chemotherapeutic drugs (cyclophosphamide, vincristine, therarubicin, and cisplatin), irradiation of the abdominal cavity, and surgical resection of the adrenal gland, followed by autologous peripheral blood stem cell transplantation (PBSCT). After treatment, VMA and HVA were normalized to 2.4 and 4.6 μg/mg Cr, respectively, and ^123^I-MIBG accumulation was not detected by scintigraphy.

**Figure 1:**
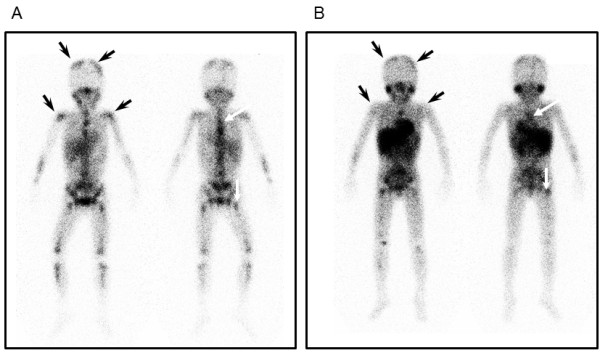
^**123**^**I-MIBG scintigrams taken before (A) and after (B)**^**131**^**I-MIBG treatment with allo-CBSCT.**^123^I-MIBG accumulation decreased after treatment in the parietal bone and humerus (black arrows), but it was still detected in the thoracic vertebrae and femur (white arrows).

The recurrence, which presented with multiple metastases in the bone marrow, occurred 2 years after PBSCT (Figure 1A). At this time, VAM and HVA had increased to 273.5 and 87.7 μg/mg Cr, respectively. After 4 cycles of chemotherapy with topotecan and etoposide, ^131^I-MIBG treatment was performed at a dose of 18 mCi/kg. We decided to perform cord blood stem cell transplantation (CBSCT) for hematopoietic rescue after the myeloablative therapies.

After injection of ^131^I-MIBG (18 mCi/kg), the patient was isolated in a shielded room for 7 days. Nine days after ^131^I-MIBG infusion, the patient was treated with busulfan (1.1 mg/kg/day, 4 times daily on days −8 to −5) and melphalan (90 mg/m^2^/day, once daily, on days −4 and −3) before cord blood stem cells with one HLA-DR locus mismatch were transfused [6]. Cyclosporine-A and methylprednisolone were administered as a prophylaxis against acute GVHD. Although the patient developed grade II acute GVHD with skin erythema which was controlled additional predonisolone, no other significant complications occurred. The patient’s neutrophil count was >500 /μl at 26 days and the platelet count was >20,000 /μl at 35 days after CBSCT. VMA and HVA were normalized to 22.6 and 10.1 μg/mg Cr, respectively. ^123^I-MIBG accumulation was significantly decreased in parietal bone and humerus (Figure 1, black arrows); however, ^123^I-MIBG was still detected in the thoracic vertebrae and femur after CBSCT (Figure 1B, white arrows). Unfortunately, the patient died 12 months after CBSCT, even though VAM and HVA were within the normal ranges for 5 months.

## Discussion

Because MIBG is selectively concentrated in sympathetic nervous tissue, ^131^I-MIBG tends to accumulate in neuroblastoma cells [4,5]. Thus, ^131^I-MIBG is potentially capable of selectively delivering a substantial radiation dose to neoplastic cells while sparing normal tissues. In our patient, the number of residual neuroblastoma cells decreased after ^131^I-MIBG treatment and CBSCT.

Allo-SCT for the treatment of neuroblastoma is considered an alternative method when autologous stem cells cannot be harvested in sufficient quantity. The superiority of allo-SCT to auto-SCT has not been clearly demonstrated [4,7], although some studies have reported a graft-versus-tumor (GVT) effect in patients with advanced neuroblastoma [7,8]. We believe that although ^131^I-MIBG treatment combined with allo-CBSCT megatherapy did not induce complete remission, the normalization of VMA and HVA, which lasted for 5 months, and the prolonged survival for 12 months were due to the reduction of neuroblastoma cells by ^131^I-MIBG treatment together with a GVT effect.

Our patient experienced relapse in the bone marrow 2 years after auto-PBSCT, suggesting that some minimal residual disease remained, even though VMA and HVA were within normal ranges, and the accumulation of ^123^I-MIBG was not observed. Chemotherapy alone has been reported to yield unsatisfactory results when used to target neuroblastoma cells in the bone marrow [9,10]. Thus, complete elimination of minimal residual neuroblastoma is an important therapeutic goal. ^131^I-MIBG and GVT could target neuroblastoma cells by different mechanisms; therefore, they could be synergistically effective against minimal residual disease. Based on our experience with this patient, we propose that ^131^I-MIBG treatment combined with allo-SCT may be an effective first-line therapy for high-risk neuroblastoma (Stage 4 or MYCN amplification); however, the cost and availability of equipment to perform ^131^I-MIBG treatment may be prohibitive for some institutions.

## Conclusion

Treatment of ^131^I-MIBG with allo-SCT was effective and safe for high-risk neuroblastoma.

### Consent

Written informed consent was obtained from the parents of the patient for publication of this Case report and any accompanying images. A copy of the written consent is available for review by the Editor-in-Chief of this journal.

## Competing interests

The authors declare that they have no competing interests.

## Authors’ contributions

All authors have equally participated in drafting of the manuscript and/or critical revision of the manuscript for important intellectual content. All authors read and approved the final manuscript.
